# Sodium arsenite-induced changes in the wood of esca-diseased grapevine at cytological and metabolomic levels

**DOI:** 10.3389/fpls.2023.1141700

**Published:** 2023-04-11

**Authors:** Sophie Trouvelot, Christelle Lemaitre-Guillier, Julie Vallet, Lucile Jacquens, Antonin Douillet, Mourad Harir, Philippe Larignon, Chloé Roullier-Gall, Philippe Schmitt-Kopplin, Marielle Adrian, Florence Fontaine

**Affiliations:** ^1^ Agroécologie, Centre National de la Recherche Scientifique (CNRS), Institut National de Recherche pour l'agriculture, l'alimentation et l'environnement (INRAE), Institut Agro Dijon, Univ. Bourgogne, Univ. Bourgogne Franche-Comté, Dijon, France; ^2^ Université de Reims Champagne-Ardenne, Unité de recherche Résistance Induite et Bioprotection des Plantes (RIBP) USC Institut National de Recherche pour l'agriculture, l'alimentation et l'environnement (INRAE) 1488, Reims, France; ^3^ Research Unit Analytical BioGeoChemistry, Helmholtz Munich, Neuherberg, Germany; ^4^ Chair Analyt Food Chem, Technical University Munich, Freising, Germany; ^5^ Institut Français de la Vigne et du Vin (IFV) Pôle Rhône-Méditerranée, Rodilhan, France

**Keywords:** Chardonnay, trunk diseases, metabolites, toxins, histology, autofluorescence, vineyard, plant defenses

## Abstract

In the past, most grapevine trunk diseases (GTDs) have been controlled by treatments with sodium arsenite. For obvious reasons, sodium arsenite was banned in vineyards, and consequently, the management of GTDs is difficult due to the lack of methods with similar effectiveness. Sodium arsenite is known to have a fungicide effect and to affect the leaf physiology, but its effect on the woody tissues where the GTD pathogens are present is still poorly understood. This study thus focuses on the effect of sodium arsenite in woody tissues, particularly in the interaction area between asymptomatic wood and necrotic wood resulting from the GTD pathogens’ activities. Metabolomics was used to obtain a metabolite fingerprint of sodium arsenite treatment and microscopy to visualize its effects at the histo-cytological level. The main results are that sodium arsenite impacts both metabolome and structural barriers in plant wood. We reported a stimulator effect on plant secondary metabolites in the wood, which add to its fungicide effect. Moreover, the pattern of some phytotoxins is affected, suggesting the possible effect of sodium arsenite in the pathogen metabolism and/or plant detoxification process. This study brings new elements to understanding the mode of action of sodium arsenite, which is useful in developing sustainable and eco-friendly strategies to better manage GTDs.

## Introduction

Viticulture is of great economic importance worldwide with an estimated area of 7.3 million hectares and a market of nearly 30 billion euros (OIV, April 2020, https://oiv.int/). However, it faces major problems including climate change and protection against diseases that affect yield, wine quality, and the safeguarding of vineyards. Most cryptogamic diseases are controlled by fungicides, some of which are known for their health and environmental risks as recently reported by Dumitriu Gabur et al. (2022). Related to these risks, some fungicides are prohibited, leaving ineffective solutions available for some diseases, especially grapevine trunk diseases (GTDs). These diseases were controlled by a pesticide based on sodium arsenite, banned in 2001 in France and 2003 in other European countries (Spinosi et al., 2009). This pesticide was strongly effective against esca disease, one of the three main GTDs with *Botryosphaeria* dieback and *Eutypa* dieback (Larignon et al., 2008; Larignon, 2016). These GTDs are considered to be the most destructive grapevine diseases in the world, leading to an alteration of vineyard heritage and serious economic losses in the wine industry (De la Fuente et al., 2016). The estimate of their incidence over 6 years reaches more than 10% of the 329 French studied vineyards for both esca disease and *Botryosphaeria* dieback and 25% for *Eutypa* dieback (Bruez et al., 2013). Considering a replacement of only 1% of plants per year, the overall annual global financial cost was estimated in 2012 at 1.132 billion euros, for example (Hofstetter et al., 2012).

Esca disease is a complex of diseases caused by several fungi, such as *Phaeoacremonium minimum*, *Phaeomoniella chlamydospora*, and *Fomitiporia mediterranea* (Surico et al., 2008; Surico, 2009). These pathogens are localized in the woody tissues of perennial organs and, to a lesser extent, in lignified 1-year-old canes but absent in the leaves where symptoms are expressed (Larignon and Dubos, 1997; Graniti et al., 2000; Fourié and Halleen, 2002; Retief et al., 2006; Bortolami et al., 2021). Leaf external symptoms consist of a chronic form characterized by the appearance of typical tiger-like necrosis and chlorosis. Apoplectic form turns to sudden wilting of leaves followed by rapid death of one or more canes or even the entire plant (Mugnai et al., 1999; Mondello et al., 2018a). The most common wood symptoms include degradation, namely, white rot and multiple discoloration patterns, such as i) black streaks in the wood involving one or more xylem vessels and ii) areas of brown and dark necrosis circumscribing the pith, which is most frequently observed (Larignon and Dubos, 2001; Lecomte et al., 2012; Mondello et al., 2018a).

Until 2003 in European countries, GTDs, especially esca disease, were limited in vineyards by the use of sodium arsenite (Spinosi et al., 2009). Since the banning of this fungicide, due to its high toxicity to human health (Saha et al., 1999; Spinosi et al., 2009), no effective treatment is now available to control them. Other chemical compounds such as triazoles (Dula et al., 2007; Gramaje et al., 2009) or Fosetyl-Al^®^ (Di Marco et al., 2011; Díaz and Latorre, 2013) have been tested, but their effectiveness depends in particular on the application mode, location (nursery or vineyard), and the targeted pathogen (for review, see Gramaje et al., 2018; Mondello et al., 2018a). Recently, a copper-based product combined with a carrier, hydroxyapatite, and plant extracts have been evaluated (Battiston et al., 2021; Mondello et al., 2021; Reis et al., 2021; Mondello et al., 2022; Reis et al., 2022) as well as aqueous ozone (Romeo-Oliván et al., 2021). Meanwhile, preventive practices such as delayed pruning and application of pruning-wound protectants (Weber et al., 2007; Rolshausen et al., 2010), and “curative” practices such as trunk surgery by removing necrotic wood (Cholet et al., 2021; Pacetti et al., 2021) and regrafting are being developed (for review Mondello et al., 2018b). Finally, biocontrol strategies including the use of species of *Trichoderma* (Di Marco et al., 2021; Leal et al., 2021), *Pythium oligandrum* (Gerbore et al., 2013; Yacoub et al., 2016; Yacoub et al., 2020), and *Bacillus subtilis* or other bacteria (Halleen et al., 2010; Haidar et al., 2016; Pinto et al., 2018; Leal et al., 2021) are also being investigated and are still in progress. However, to date, all these strategies remain insufficient to control GTDs, especially esca disease, compared to sodium arsenite. Therefore, a better understanding of the sodium arsenite actions is needed to provide alternative eco-friendly solutions to prevent or limit GTD development and incidence.

Previous studies on the mode of action of sodium arsenite are rather limited and mainly focused on its impact on GTD pathogens (Da Costa, 1971; Carbonell-Barrachina et al., 1997; Larignon et al., 2008) and dynamics *in planta* after application (Carbonell-Barrachina et al., 1997; Larignon et al., 2008). Between 2013 and 2016, a global project dedicated to the characterization of the action of sodium arsenite was carried out in order to propose a mimic strategy against GTDs, of similar effectiveness but with lower environmental risks. This project particularly focused on the impact of sodium arsenite on i) the wood microbiota including GTD pathogens and ii) plant physiology at cytological, transcriptomic, and metabolomic levels. Regarding wood microbiota, Bruez et al. (2020) reported a stronger effect of sodium arsenite treatment on the fungal community compared to the bacterial one and highlighted its effectiveness, especially against *F. mediterranea*—an esca pathogen responsible for the necrotic white-rot tissues (Mugnai et al., 1999). On grapevine physiology, Songy et al. (2019) observed first a decrease and then stimulation of photosynthesis with simultaneous activation of some grapevine defense responses. The objective of this paper focuses on the effect of sodium arsenite in woody tissues, particularly in the interaction area between healthy wood and necrotic wood resulting from the GTD pathogens’ activities. A combination of global and targeted approaches was used: metabolomics to obtain a metabolite fingerprint of sodium arsenite treatment and microscopy to visualize its effects at the histo-cytological level. Overall, these studies will provide useful insights into the process of protection by sodium arsenite against GTDs.

## Materials and methods

### Plant material

In 2013, spotting was performed in a 27-year-old vineyard of cv. Chardonnay grafted on SO4 rootstock planted at 7,575 plants/ha. Plants were vertically trained and pruned according to the Chablis method. The vineyard is located in the province of Epernay (Avize, France, GPS coordinates: 48°58′29″N, 04°00′46″E) and is owned by the professional school “Lycée VitiCampus”. It is characterized by an average annual temperature of 10.8°C and 480 mm of annual precipitation, and the soil is clay and sandy loam. No treatment with sodium arsenite has been performed in this experimental plot in the past. Fifteen plants were selected and labeled: five were apparently healthy, without foliar esca disease symptoms, and 10 were diseased, with esca disease chronic foliar symptoms as described by Mugnai et al. (1999) and Mondello et al. (2018a). Among the latter, five were treated the next winter year (2014) with sodium arsenite. For both conditions, treated and not treated by sodium arsenite, a classical conventional phytosanitary itinerary was applied against downy mildew and powdery mildew. For the general diffusion of esca, the annual incidence was very low and close to 2% as globally observed in the Champagne vineyard (Mondello et al., 2022).

### Treatment and sampling

The following year (2014), five plants that were chronically diseased in 2013 were selected and treated with sodium arsenite (Pyralesca RS) at 1,250 g/hL until streaming, at the end of winter after pruning (10 March 2014), and before bud bursting, BBCH 00 (Meier, 2001).

**Figure 1:**
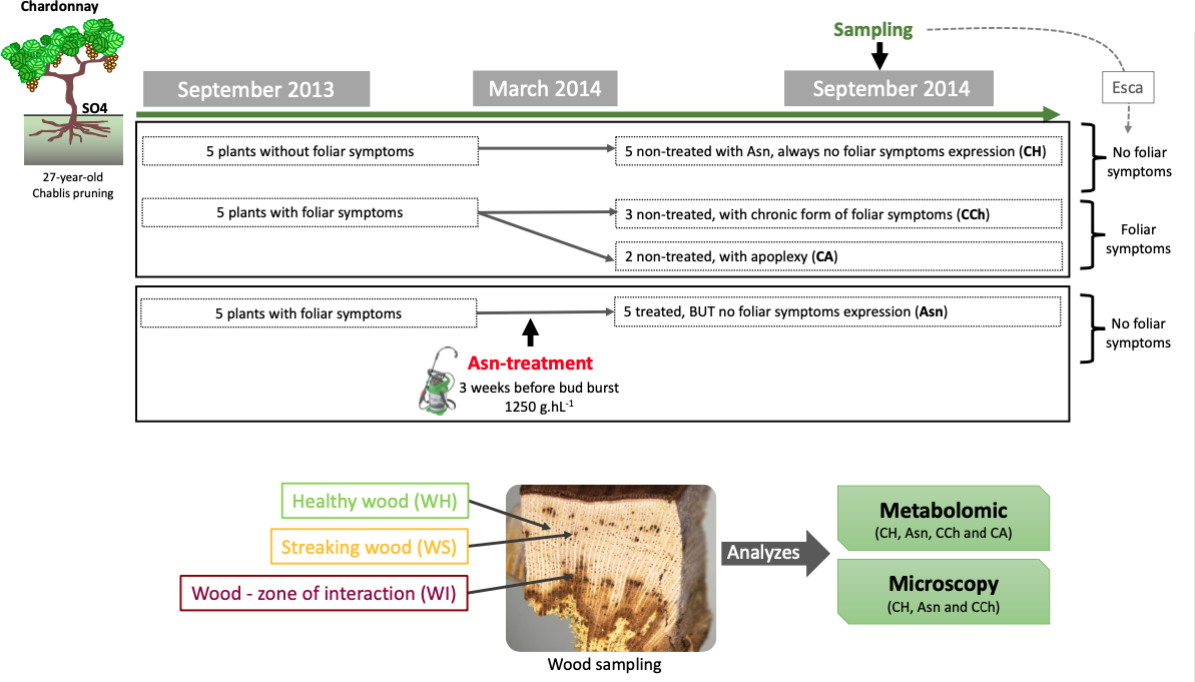
Experimental design of the study carried out in 2013–2014, including the description of the sodium arsenite treatment and the samples collected for both approaches, metabolomic and microscopy.

In the middle of September 2014, the labeled vines were divided into four groups (**Figure 1**; **Supplementary Material**): i) “Asn” for diseased vines in 2013 treated with sodium arsenite (Asn) and without external symptom expression in September 2014; ii) “CCh” for diseased vines in 2013 not treated with sodium arsenite (as control, C) and with chronic (Ch) form of esca expression in 2014; iii) “CA” for diseased vines in 2013 not treated with sodium arsenite (as control, C) and with apoplectic (A) form of esca expression in 2014; and iv) “CH” for apparently healthy vines not treated with sodium arsenite (as control, C) and visually healthy (H) in both 2013 and 2014 (**Figure 1**). In addition, the leaves of CH and Asn samples were asymptomatic of esca, whereas CCh and CA leaves were symptomatic. All vines were uprooted 1 week before harvest, and, for each vine, the wood of the trunk was collected and divided into three sample types: visually healthy area (WH), streaked area (WS), and interaction area (WI) covering unaltered and altered wood tissues (**Figure 1**). As much as possible, the samples of the different modalities were taken from equivalent wood strata, the most peripheral possible (i.e., where functional healthy wood could be found).

For metabolomics, WH, WS, and WI wood samples were immediately frozen with liquid nitrogen after being cut at the laboratory and subsequently stored at −80°C. For each modality, two and three independent vines (for CA and Asn, and CH and CCh, respectively) were sampled.

For microscopy, WH and WI samples were either i) stored at −20°C before being observed under a stereomicroscope or a scanning electron microscope or ii) sliced in pieces of strips 0.1 to 0.3 cm wide before being immediately fixed overnight at 4°C (see below). For this study, we chose not to sample the apoplectic modality (CA) for fear of reading artefactual repercussions associated with cell death of aerial parts. In this context, for each studied modality (CH, CCh, and Asn), three independent vines were sampled. For each one, a minimum of seven wood fragments were collected in each area (WS and WI).

### FTICR-MS analysis

In total, 26 wood samples (*n* = 10 WH, *n* = 7 WS, and *n* = 9 WI) were analyzed by Fourier-transform ion cyclotron resonance mass spectrometry (FTICR-MS). They were all prepared with the same protocol. Samples were ground to a fine powder in liquid nitrogen with a Mixer Mill MM 400 (Retsch, Haan, Germany) before analysis, and 15 mg of each was added with 1 ml of methanol (Liquid chromatography-mass spectrometry (LC-MS) grade, Fluka Analytical; Sigma-Aldrich, St. Louis, MO, USA). After sonication for 30 min and centrifugation (25,000 *g*, 10 min, room temperature), the supernatant was collected and re-diluted in methanol (1/50 v/v). Ultra-high-resolution mass spectra were acquired using an FTICR-MS (solariX, BrukerDaltonics GmbH, Bremen, Germany) equipped with a 12-Tesla superconducting magnet (Magnex Scientific Inc., Yarnton, UK) and an APOLO II ESI source (Bruker Daltonics GmbH, Bremen, Germany) operated in the negative ionization mode. Samples were introduced into the micro-electrospray source at a flow rate of 120 μl/h. Spectra were acquired with a time domain of 4 mega words over a mass range of 100 to 1,000, and 300 scans were accumulated per sample.

Spectra were externally calibrated on arginine clusters (10 mg/L in methanol). Further internal calibration was performed for each sample by using a list of ubiquitous fatty acids and recurrent wine compounds, allowing mass accuracies of 0.1 ppm (Gougeon et al., 2009). Exact masses were then run through the Netcalc algorithm, an in-house software tool to obtain unambiguous chemical formulas (Tziotis et al., 2011) and were validated next by setting van Krevelen chemical constraints (O/C ratio ≤ 1; double bond equivalent (DBE): 0 < DBE/C < 5 H/C ratio ≤ 2n + 2 and aromaticity index (IA): −20 < IA < 0.7; element counts: C ≤ 100, H ≤ 200, O ≤ 80, N ≤ 3, S ≤ 3 and P ≤ 1) applied to exclude rare or impossible formulas. It also enabled the classification of formulas according to their atomic compositions (i.e., CHO, CHOS, CHON, CHONS, CHOP, and CHONP CHONSP). Mass annotations were obtained by KEGG, HMDB, and LipidMaps databases queries against *Vitis vinifera* organism (0.1 ppm tolerance error value) by Masstrix software facility (https://metabolomics.helmholtz-muenchen.de/masstrix3/). The raw formula of the *m/z* was used for functional categorization of the annotated compounds (lipids, peptides, amino sugars, carbohydrates, nucleotides, phytochemicals (i.e., secondary metabolites), and NM when not matching) using the multidimensional stoichiometric constraint classification (MSCC) described by Rivas-Ubach et al. (2018), based on the C/H/O/N/P stoichiometric ratios. Perseus software version 1.6.8.0 (https://maxquant.net/perseus/) was used to determine significant compounds (ANOVA multivariate statistical analysis (*p* < 0.05)) to perform additional t-test comparisons (false discovery rate (FDR) < 0.05) between two sample groups and to draw heatmaps. The 20 most regulated annotated compounds between the two sample groups were sorted in the Top20 lists. Principal component analyses (PCAs) were performed, and illustrations were built with RStudio software (ade4 and vegan packages). Targeted fungal toxins and plant secondary metabolites were searched in the peak lists based on their theoretical *m/z* but should be considered putative (**Supplementary Table S1**).

### Cytological approaches

#### For stereomicroscopy observations

The slices of the wood fragments (1 cm side cubes) were first refreshed using a razor blade before being directly observed under a stereo microscope (SMZ25 Nikon). Those fresh samples were thus observed in a bright field and under epifluorescence (UV excitation at 330–380 nm and/or blue at 420–495 nm). In bright fields, this method made it possible to visualize the state of plant cells (healthy areas, areas obstructed by tyloses or gums, or tinder areas). Under epifluorescence, it was possible to reveal the autofluorescence of defense compounds and in particular phenolic compounds, which are excitable at the wavelengths used.

#### Scanning electron microscopy observations

The slices of the wood fragments (1 cm side and 0.5 cm thick) were first refreshed using a razor blade before being directly observed under a scanning electron microscope (Philips XL-30 ESEM LaB6). This method allowed us to characterize the wood surfaces.

In addition, in order to detect some traces of Asn in the tissues observed, the samples were analyzed using a scanning microscope (JEOL JSM 7600F) coupled with an X-ray detector (EDX 80 mm X-Max, Oxford Instruments, Abingdon, UK). X-ray microanalysis allows elemental analysis by detecting the characteristic X-rays of the elements present. It allows in particular specific analyses with a spatial resolution of the order of 1 μm^3^ and is qualitative as well as quantitative. In this sense, it is able to detect Asn of approximately 50 ppm.

All these observations were made at the DImaCell platform of the UMR Agroecology (INRAE, Université Bourgogne Franche-Comté, Dijon, France).

#### Light microscopy observations of semi-thin sections

Wood samples were first fixed overnight at 4°C in 0.1% glutaraldehyde/4% paraformaldehyde prepared in 0.1 M of phosphate buffer with pH 7.2 and supplemented with 1% sucrose and 0.1% Tween 20. Then, samples were dehydrated by successive baths in ethanol (30%, 30 min at 4°C; 50%, 1 h at −20°C; 70%, 1 h at −20°C; 95%, 30 min at −20°C; and 100%, 30 min at −20°C). The samples were then embedded in LR White resin (London Resin Company, London, UK) and underwent polymerization in Beem^®^ capsules (Hemi-hyperbole beem capsule, Agar Scientific Limited, Stansted, UK) under UV at −20°C, as described in Trouvelot et al. (2008). Semi-thin transverse sections (500 nm thick) were then made using an UltraCut E ultra-microtome (Reichert-Jung) equipped with a “histo” diamond (6 mm, 45°). The semi-thin sections were placed on glass slides (76 × 26 mm, Knittel Glass; approximately 20 sections per slide) before being either i) stained with toluidine blue (1% in aqueous solution) or ii) observed directly by epifluorescence. For each treatment, seven to nine distinct samples (i.e., woody fragments embedded in resin blocks) were studied, and at least two distant areas were observed per block.

## Results and discussion

According to the first observations, the plants treated with sodium arsenite (Asn) did not express esca foliar symptoms, the five apparently healthy (CH) plants in 2013 retained the same status in 2014, and the five plants expressing esca symptoms (CCh) in 2013 re-expressed them in 2014, with three in chronic form and two apoplectic. The non-expression of esca foliar symptoms of diseased plants after sodium arsenite treatment confirms the healing and efficacy of this treatment against esca (Larignon et al., 2008; Songy et al., 2019; Del Frari et al., 2022).

### Wood samples discriminate between sodium arsenite-treated and untreated grapevines

**Figure 2:**
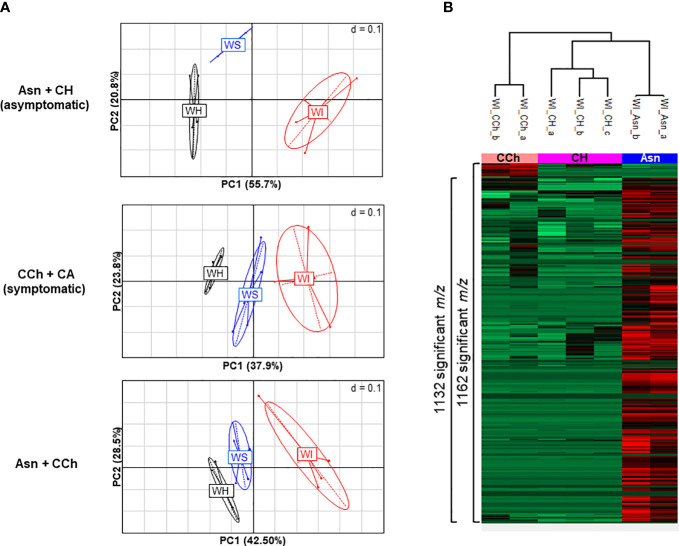
**(A)** PCA illustrations of cross-comparisons of wood samples: asymptomatic Asn+CH (top), symptomatic CCh+CA (middle), and Asn+CCh (bottom). Two to four replicates per wood area (healthy area, WH; streaked area, WS; and interaction zone, WI) per sample group (sodium arsenite-treated, Asn; healthy, CH; symptomatic expressing chronic disease, CCh; and apoplectic, CA). **(B)** Metabolite variations in wood interaction zone (WI) of significant 1,173 *m/z* (*p* < 0.05) between healthy (CH), chronic disease form (CCh), and sodium arsenite-treated grapevines (Asn). Numbers of significant t-test *m/z* (FDR < 0.05) that discriminate pair-to-pair groups are indicated on the side of the graph. PCA, principal component analysis; FDR, false discovery rate.

Principal component analyses of all wood samples (4,825 *m/z*) of asymptomatic (Asn and CH) and symptomatic vines (CCh and CA) were performed and compared (**Figure 2**). It showed a clear separation of healthy wood (WH) from streaked wood (WS) samples and even further from the interaction area (WI) for the three comparisons (**Figure 2A**). Focus was therefore placed on the wood of the interaction zone (WI), and the hierarchical clustering analysis confirmed that this subgroup allowed clear discrimination between Asn-treated grapevines and the other two, CCh and CH, by 1,132 and 1,162 *m/z*, respectively (**Figure 2B**).

The treatment by sodium arsenite therefore strongly and locally modified the metabolome of the interaction zone, which is the intermediate area between the healthy wood (WH), not yet infected by GTD pathogens, and the infected necrotic central wood. The interaction area by its location may have a role in limiting the colonization of GTD pathogens and perhaps in controlling the expression of GTD symptoms. A thorough characterization of the response in this particular area was performed.

### Sodium arsenite induced significant changes in the metabolome of the interaction zone

Statistical analysis was performed on WI samples to compare Asn, CCh, and CH groups. Analyses were first applied to all *m/z* data sets; characterizations (formula and functional categorizations) and annotations were then carried out if significant (*p* < 0.05) *m/z* (**Supplementary Table S2**). Throughout the analysis process, the output lists were up to 98% identical.

**Figure 3:**
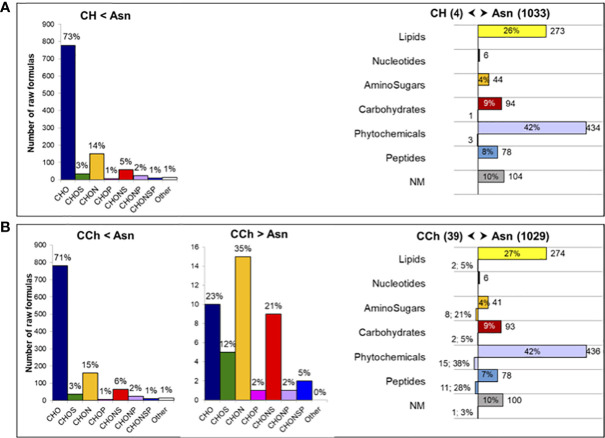
Comparison of the metabolome of the wood interaction area of vines treated or not by sodium arsenite. Comparison of the significantly regulated *m/z* of **(A)** healthy (CH) and Asn-treated (Asn) vines and **(B)** diseased (CCh) and Asn-treated (Asn) vines. Chemical classes of the corresponding raw formulas containing C, H, O, N, S, and P atoms (determined from 1,057 and 1,087 *m/z*), with the indication of the percentage of distribution in each class (left panels). Functional categorization (right panels) of the annotated compounds differently regulated in samples (1,037 and 1,068 compounds), with the indication of the number and percentage of distribution in each biochemical category. Functional categories were predicted from raw formulas according to Rivas-Ubach et al. (2018). NM, non-matched.

Although vines treated with sodium arsenite did not express leaf symptoms, the metabolome in the wood interaction area (WI-Asn) was quite different from that of the WI-CH group. The comparison between WI-Asn and WI-CH samples highlighted 1,132 *m/z* accumulated significantly differently (**Supplementary Table S2**). These *m/z* values led to the creation of a list of 1,071 raw formulas, of which 1,057 were chemically classified into compounds CHO (73%), CHON (14%), and CHONS (5%) (**Figure 3A**). The overall analysis ended with 1,037 unique raw formulas—when removing isotopic forms and adduct ions (**Supplementary Table S2**)—among which 1,033 were more accumulated in WI-Asn compared to WI-CH and four were less accumulated in WI-Asn than in WI-CH (**Figure 3A**). They were mainly phytochemicals (42%) and lipid-like (26%). From the 1,037 obtained raw formulas, 546 compounds could be identified in databanks. Among them, the Top20 most accumulated annotated compounds in WI-Asn samples were predicted to be phytochemicals (60%), lipids (20%), and carbohydrates (20%) (**Supplementary Table S3A**).

The metabolome of WI-Asn was also different from that of WI-CCh (**Figure 2B**). The comparison between these two sample sets highlighted 1,162 *m/z* significantly regulated, of which 1,121 were common to the comparison Asn *versus* CH (**Supplementary Table S2**). Of these, 1,087 *m/z* (distributed as 43 more accumulated in WI-CCh than in WI-Asn and 1,044 more accumulated in WI-Asn than in WI-CCh) were classified according to their raw formula. Those accumulated in WI-Asn (compared to WI-CCh) were mainly composed of CHO (71%), CHON (15%), and CHONS (6%), whereas those accumulated in WI-CCh (compared to WI-Asn) were mainly CHON (35%), CHO (23%), and CHONS (21%) (**Figure 3B**). Overall, it led to 1,068 unique formulas (**Supplementary Table S2**), with 39 more accumulated in WI-CCh and 1,029 more accumulated in WI-Asn (**Figure 3B**). The compounds accumulated in WI-Asn were predicted to be mainly phytochemicals (42%) and lipids (27%), while those accumulated in WI-CCh were rather phytochemicals (38%), peptides (28%), and amino sugars (21%, **Figure 3B**). Among them, the Top20 annotated compounds lesser accumulated in WI-Asn, compared to WI-CCh, were phytochemicals (60%), lipids (20%), and carbohydrates (20%). One was annotated as piceatannol, a resveratrol derivative (**Supplementary Table S3B**). Among the Top20 annotated compounds more abundant in WI-Asn, 60% were phytochemicals, 20% were lipids, and 20% were carbohydrates (**Supplementary Table S3B**). Altogether, treatment with sodium arsenite has effects on the metabolome of the wood interaction zone and mainly results in the accumulation of phytochemicals and lipids. These two categories were previously shown as a discriminant of the brown stripe of the wood of vines infected by *Botryosphaeria* dieback and the adjacent asymptomatic white wood (Lemaître-Guillier et al., 2020).

### Putative toxins are detected in all wood samples, including those of asymptomatic vines

As the GTD pathogens produce phytotoxins (Andolfi et al., 2011; Abou-Mansour et al., 2015; Cimmino et al., 2017; Masi et al., 2018; Reveglia et al., 2019; Trotel-Aziz et al., 2019), a focus on the *m/z* corresponding to putative ones (**Supplementary Table S1**) was carried out on the wood samples WH, WS, and WI of CH, CCh, CA, and Asn-treated grapevines (**Table 1**).

**Table 1:** Peak intensities of putative phytotoxins detected into wood samples (WH, WS and WI) in asymptomatic: healthy (CH) and sodium arsenite-treated (Asn), and symptomatic: chronic form of disease (CCh) and apoplectic (CA) grapevines.

Fungal toxins	CH	Asn	CCh	CA
Scopoletin (×10^7^)	2.83	2.58	2.17	1.49
± Std	2.62	2.64	2.44	1.11
6-Methoxymellein (×10^7^)	1.55	2.02	1.78	1.43
± Std	0.73	1.46	0.77	0.47
OH-Mellein (scytalone) (×10^6^)	6.96	7.82	6.39	6.47
± Std	3.63	4.79	3.04	2.63
Resveratrol-sulfate (×10^7^)	1.08	0.72	1.15	1.09
± Std	0.98	0.76	1.27	0.71
OH-Tyrosol (×10^5^)	2.69	0.00	5.82	3.16
± Std	6.59	0.00	8.92	7.74
OH-Tyrosol 1-*O*-glucoside (×10^8^)	1.00	1.13	0.99	1.20
± Std	0.18	0.73	0.39	0.71
*cis*-4-Hydroxy-scytalone (×10^7^)	1.23	2.21	1.52	2.57
± Std	0.34	1.30	0.37	1.25
Dimethylallyl-scopoletin (×10^6^)	3.60	3.08	3.09	5.66
± Std	0.63	2.95	0.73	3.18
Terremutin (×10^6^)	0.81	1.11	0.57	2.54
± Std	0.89	1.02	0.87	2.46
Tyrosol 4-sulfate (×10^6^)	1.23	1.01	2.01	2.19
± Std	2.17	1.52	1.85	1.20
Mellein (×10^6^)	1.64	1.41	2.10	2.14
± Std	1.41	2.04	1.31	0.36

Colored gradient indicates intensities averaged levels (scale folds indicated in brackets). Std: standard deviation per wood sample group (CH: n=6, Asn: n=6; CCh: n=8 and CA: n=6).

Putative toxins were detected with different abundances, ranging from 10^5^ for OH-tyrosol to 10^8^ for OH-tyrosol 1-*O*-glucoside, in the different wood sample groups (**Table 1**). Indeed, some of them were detected preferentially in the wood of asymptomatic CH and Asn vines, compared to those of symptomatic vines, such as scopoletin, 6-methoxymellein, and OH-mellein. Six other toxins were rather more detected in the wood of apoplectic vines such as OH-tyrosol 1-*O*-glucoside, *cis*-4-hydroxy-scytalone, dimethylallyl-scopoletin, terremutin, tyrosol-4-sulfate, and mellein. Mellein and terremutin are especially produced by Botryosphaeriaceae species such as *Neofusicoccum parvum*, *Diplodia seriata*, or *Lasiodiplodia* (Andolfi et al., 2011; Abou-Mansour et al., 2015; Cimmino et al., 2017; Reveglia et al., 2019; Reveglia et al., 2021; Trotel-Aziz et al., 2022), and their detection in woody tissues was first reported by Abou-Mansour et al. (2015) in the brown-striped wood of *Botryosphaeria* dieback-diseased vines. Our results, therefore, are consistent with those of Bruez et al. (2021), who reported that these esca-expressing vines were colonized by Botryosphaeriaceae species.

Putative toxins could be found in the healthy (CH) and esca-diseased vines developing the chronic form (CCh). As discussed by Del Frari et al. (2022), the role of toxins in explaining the occurrence and yearly fluctuations of leaf stripe symptoms is not always obvious: wood degradation by-products and microbe-induced metabolites probably also play a key role (Mugnai et al., 1999; Bruez et al., 2020; Schilling et al., 2021; Del Frari et al., 2022). Our results also further suggest that in the apoplexy process, which is a more pronounced expression than the chronic form, there is a higher amount of toxins produced by GTD fungi. Similar observations were described by Magnin-Robert et al. (2016), who reported significant production of (3*R*,4*R*)-hydroxymellein in the green stem, cordon, and trunk of esca-apoplectic vines compared to esca-chronic and asymptomatic grapevines. Toxins are one of the three possible factors triggering the leaf stripe symptoms (Mugnai et al., 1999).

The next biological question was as follows: how can we explain the accumulation of some phytotoxins in the wood of vines treated with sodium arsenite and the no-emergence of foliar symptoms? Could this mean that sodium arsenite does not directly “kill” fungi? The hypothesis could be that sodium arsenite, as described by Songy et al. (2019), induced physiological changes in leaves and/or other organs, such as the green stem (Fontaine F., personal communication), that could counteract the negative effect of phytotoxins and finally avoid the foliar symptom expression. Moreover, Bruez et al. (2021) reported that sodium arsenite treatment strongly impacts the fungal microbiota and observed, among other things, a strong reduction in the abundance of *F. mediterranea* and an unexpected increase in the abundance of *P. chlamydospora* occurring at the borders of wood symptoms. They hypothesized that arsenite treatment contributes to making the host wood area colonizable again, providing newly available nutrients for the endophytic microbiota. Del Frari et al. (2022) also proposed the host vascular-based transport hypothesis of leaf stripe symptom-inducing molecules (LSSIMs) to explain the development of leaf stripe symptoms. They suggested that sodium arsenite could indirectly reduce LSSIMs and thus contribute to preventing symptom expression.

### Some relevant secondary metabolites are in higher abundance in the wood of vines treated by sodium arsenite

Similarly, plant secondary metabolites (**Supplementary Table S1**) were searched in sample peak lists. We particularly focused on stilbene phytoalexins (resveratrol, the resveratrol-glucoside piceid, oxy-resveratrol, dihydro-resveratrol, epsilon-viniferin, delta-viniferin, and astringin), flavonoids (quercetin-*O*-glucuronide and daidzein), phytohormones (salicylic acid and methyl salicylic acid), and precursors of tanins (galloyl-glucose) and lignins (caffeic acid and caftaric acid). Most of the stilbene compounds (**Table 2**) and other compounds, namely, phenolic compounds, salicylic acid, and methyl salicylate (**Table 3**), were detected with more intensity in WI-Asn samples than in the others. Piceid and astringin were rather found in apoplectic CA wood samples. Stilbenes are well-known grapevine phytoalexins, and some of them have a high antimicrobial activity (Jeandet et al., 2002; Chong et al., 2009; Adrian et al., 2012; Lambert et al., 2012). They can be induced by microbial colonization or by toxins (Abou-Mansour et al., 2015; Rusjan et al., 2017; Stempien et al., 2017; Trotel-Aziz et al., 2019). Del Frari et al. (2022) suggested that sodium arsenite could improve the vines’ tolerance to LSSIMs by controlling the vines’ physiology and defense response. In this way, most of the putative toxins tend to be found in lower amounts in Asn vines, concomitant with a higher abundance of some stilbenes and other secondary metabolites, which ultimately leads to asymptomatic vines, contrary to what was observed in CA vines, leading to the emergence of the severe form of GTDs. Overall, these putative toxin results enhance the involvement of these toxic compounds in the GTD foliar symptom expression as summarized in the conceptual model proposed by Claverie et al. (2020).

**Table 2:** Intensities of targeted stilbenes secondary metabolites in wood samples (WH, WS and WI) in asymptomatic: healthy (CH) and sodium arsenite-treated (Asn) and symptomatic: chronic form of disease (CCh) and apoplectic (CA) grapevines.

Stilbenes	CH	Asn	CCh	CA
Resveratrol (×10^10^)	3.38	3.16	1.60	0.74
± Std	3.76	4.15	2.73	0.67
Ellagic acid (×10^8^)	3.44	2.87	1.35	0.74
± Std	4.48	3.66	2.87	0.89
Methyl-resveratrol-glucoside (×10^7^)	1.47	1.16	0.90	1.32
± Std	1.97	0.95	0.27	0.85
epsilon-Viniferin (×10^9^)	3.69	7.18	2.60	4.82
± Std	4.30	9.02	2.36	3.91
Dihydro-resveratrol (×10^8^)	8.31	10.78	3.59	2.59
± Std	9.19	14.03	6.45	2.45
Oxy-resveratrol (×10^8^)	7.59	8.72	4.75	4.94
± Std	8.47	10.43	6.01	3.51
delta-Viniferin-glucoside (×10^7^)	1.99	3.84	1.36	2.46
± Std	2.31	4.71	1.47	1.86
Resveratrol-*O*-glucuronide (×10^6^)	5.47	9.11	4.64	5.41
± Std	4.46	10.43	4.06	3.82
Resveratrol-galloylglucoside (×10^6^)	6.16	6.71	3.36	1.47
± Std	6.41	3.26	5.07	3.59
epsilon-Viniferin-diglucoside (×10^6^)	0.00	1.82	0.41	1.30
± Std	0.00	1.72	1.22	2.05
Resveratrol-glucoside-sulfate (×10^6^)	0.70	1.48	1.67	1.54
± Std	1.08	1.41	1.31	1.75
Resveratrol-glucoside (Piceid) (×10^9^)	1.67	2.09	1.99	2.52
± Std	0.60	0.78	0.57	1.44
Astringin (×10^8^)	1.24	1.37	1.42	1.55
± Std	0.36	0.50	0.30	0.57

Colored gradient indicates intensities averaged levels (scale folds indicated in brackets). Std: standard deviation per wood sample group (CH: n=6, Asn: n=6; CCh: n=8 and CA: n=6).

**Table 3:** Amounts of targeted other secondary metabolites in wood samples (WH, WS, and WI) in asymptomatic (healthy (CH) and sodium arsenite-treated (Asn)) and symptomatic (chronic form of the disease (CCh) and apoplectic (CA)) grapevines.

Others	CH	Asn	CCh	CA
Linoleic acid (×10^9^)	4.75	3.06	2.21	1.64
± Std	5.00	3.38	4.23	1.85
Galloyl-glucose (×10^9^)	6.49	7.70	5.01	5.26
± Std	3.10	3.64	1.64	1.84
Caftaric acid (×10^8^)	0.40	1.71	0.29	0.88
± Std	0.18	2.19	0.10	0.52
Quercetin (×10^7^)	4.41	4.79	2.84	3.12
± Std	4.56	5.69	5.32	3.53
Quercetin-*O*-glucuronide (×10^7^)	0.97	1.31	0.93	1.20
± Std	0.53	0.54	0.42	0.33
Caffeic acid (×10^7^)	0.69	1.09	0.74	1.06
± Std	0.22	0.55	0.14	0.27
Daidzein (×10^6^)	3.20	4.13	2.07	2.76
± Std	2.29	5.66	1.68	1.63
Salicylic acid (×10^6^)	2.41	5.81	2.85	3.87
± Std	0.47	1.21	1.07	1.22
Methyl salicylate (×10^6^)	1.17	2.41	1.62	1.51
± Std	1.32	1.18	0.98	1.87

The colored gradient indicates intensity averaged levels (scale folds indicated in brackets).

Std, standard deviation per wood sample group (CH, n = 6; Asn, n = 5; CCh, n = 9; CA, n = 6).

### Sodium arsenite did not modify the wood surface aspect in esca-diseased grapevine

**Figure 4:**
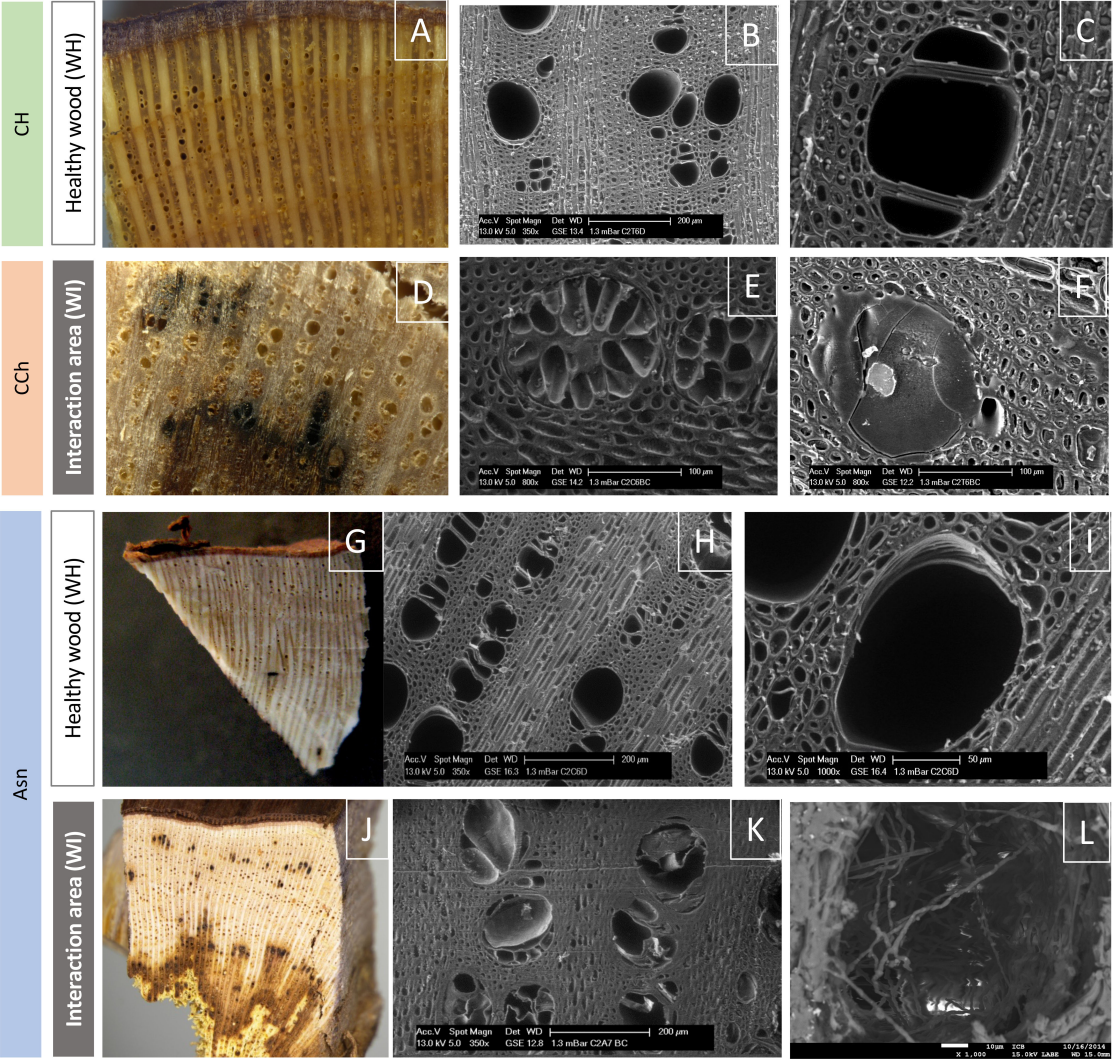
Observation of the surface of healthy woody tissues (WH) or of the interaction area (WI) by macroscopy (color photographs **A, D, G, J**) or by scanning electron microscopy (grayscale photographs **B, C, E, F, H, I, K, L**). Regardless of the modality observed (CH, CCh, and Asn), healthy woods have the same appearance **(A–C, G–I)**. In the same way, it is not possible to discriminate the modalities according to the appearance of the interaction area **(D–F, J–L)**. In this area, we observed, whatever the modality, vessels obstructed by tyloses **(E, K)** and/or gums **(F)**, as well as the presence of hyphae **(L)** approaching the zone of white-rot necrosis (amadou).

In order to determine whether sodium arsenite could have repercussions on the structuring of woody tissues, we first analyzed the surface of woody samples taken from vines affected by esca and treated or not with sodium arsenite. In the WI, the wood surface appeared similar between the samples treated (Asn) and not treated (CH and CCh) with sodium arsenite (**Figure 4**). Overall, the observed results reflected the repercussions of fungal activities in woody cells, consistent with what had already been reported in other studies (Scheck et al., 1998; Pouzoulet et al., 2014). Indeed, in these WI (brown in color), the presence of tyloses and gums obstructing the vessels was observed. This probably reflected a defensive reaction of the xylem cells in response to their colonization by vascular fungi (Yadeta and Thomma, 2013, for review; Kassemeyer et al., 2022). These observations are consistent with the presence of stilbenes reported above. Consequently, the circulation of raw sap was significantly reduced. Moreover, whatever the treatment, the closer we were to symptomatic areas of white rot characterized by wood degradation, the more we observed the presence of fungal hyphae inside the vessels. It also suggested that these fungi were not directly affected by sodium arsenite, at least in their hyphal structure. At this resolution, it was then not possible to discriminate the wood esca-diseased samples treated with sodium arsenite from the untreated samples.

In order to determine whether, in the modalities treated with Asn, we could detect and quantify arsenic, we carried out observations by scanning microscopy coupled with X-ray microanalysis. Indeed, this technique makes it possible to simultaneously obtain morphological (surface images by scanning) and chemical (elemental composition) information from a sample. In this context, we revealed that in the interaction area, no trace of arsenic was detected (**Supplementary Figure S1**). The same result was obtained in the healthy tissues of Asn-treated vines (data not shown). It, therefore, seemed i) that the treatment with sodium arsenite, carried out at the beginning of March in the vineyard, did not accumulate (nor certain products resulting from its metabolism) in the secondary wood of the treated and protected samples or ii) if it was present in these tissues, it was in a quantity of less than 50 ppm (detection threshold of the X-ray detector). However, this did not exclude that an accumulation of arsenic could have taken place in other non-targeted tissue areas (tinder in particular) or even in other plant organs, leaves, or roots in particular (Larignon and Fontaine, 2018).

### Sodium arsenite induced an increase in woody tissue autofluorescence

In order to assess whether a sodium arsenite treatment could improve the defensive state of woody tissues by increasing the impregnation of cell walls with phenolic compounds, we then observed the autofluorescence (under UV and blue excitation) of the samples.

**Figure 5:**
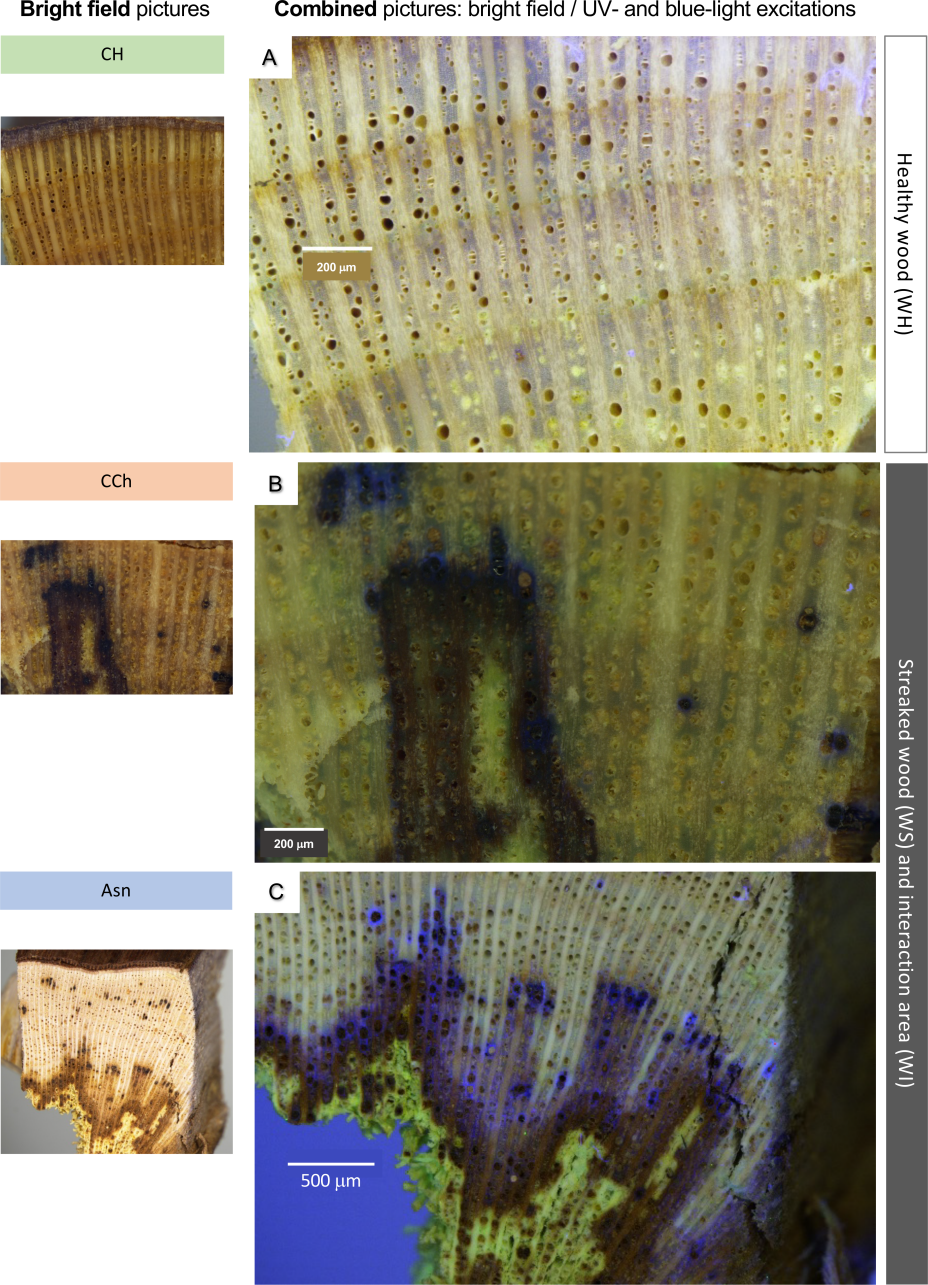
Observations, in bright-field (left pictures) and/or fluorescence microscopy **(A–C)**, of samples of woody tissues, collected on CH, CCh, or Asn vines, in healthy (WH) or interaction area (transition healthy/brown necrotic wood; WI). Bar: 200 μm **(A, B)** or 500 μm **(C)**.

Initially, the fresh samples were observed macroscopically under epifluorescence. **Figure 5** shows next to each zone observed in bright-field optical microscopy, the combined image of the color spectra (bright field), UV excitation (excitation, 359–371; emission, 397 nm), and blue excitation (excitation, 455–495 nm; emission, 505–555 nm). At this resolution, no marked autofluorescence was detected in healthy tissues of asymptomatic (**Figure 5A**) and symptomatic vines (data not shown). In contrast, in the interaction zones (WI) of esca-diseased vines not treated with sodium arsenite (CCh, **Figure 5B**), an autofluorescence signal was detected under UV excitation (dark blue autofluorescence). However, we were able to observe that this signal appeared relatively weak and very punctual. Finally, in the interaction areas of esca-diseased vines treated with sodium arsenite (Asn, WI), the same autofluorescence signal (dark blue, in response to UV excitation) could be detected but with a much higher abundance and representativeness (**Figure 5C**). This is consistent with the accumulation of secondary metabolites, especially the phenolic compounds highlighted by FTICR-MS.

**Figure 6:**
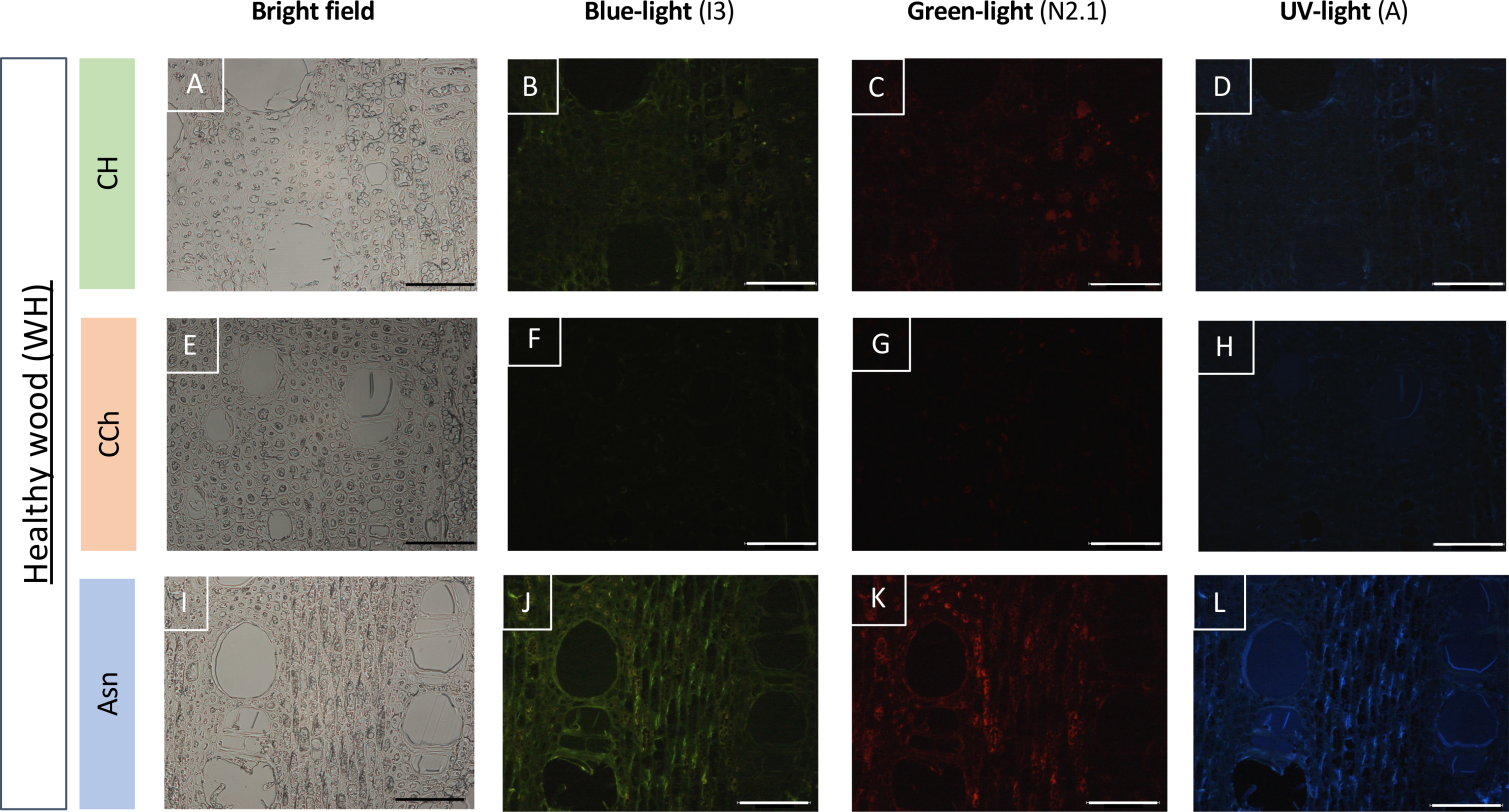
Observation of the autofluorescence of healthy woody tissues (WH) collected on CH **(A–D)**, CCh **(E–H)**, or Asn-treated vines **(I–L)** and compared according to different types of excitations (blue, green, or UV light) in epifluorescence microscopy **(B–D, F–H, J–L)**. For each modality, the observation of the same sample in bright field microscopy is given respectively in **A, E** and **I**. A slight increase in the autofluorescence signal is observed, whatever the excitation wavelength experienced, in the Asn modality **(J–L)**. Bar: 100 μm.

**Figure 7:**
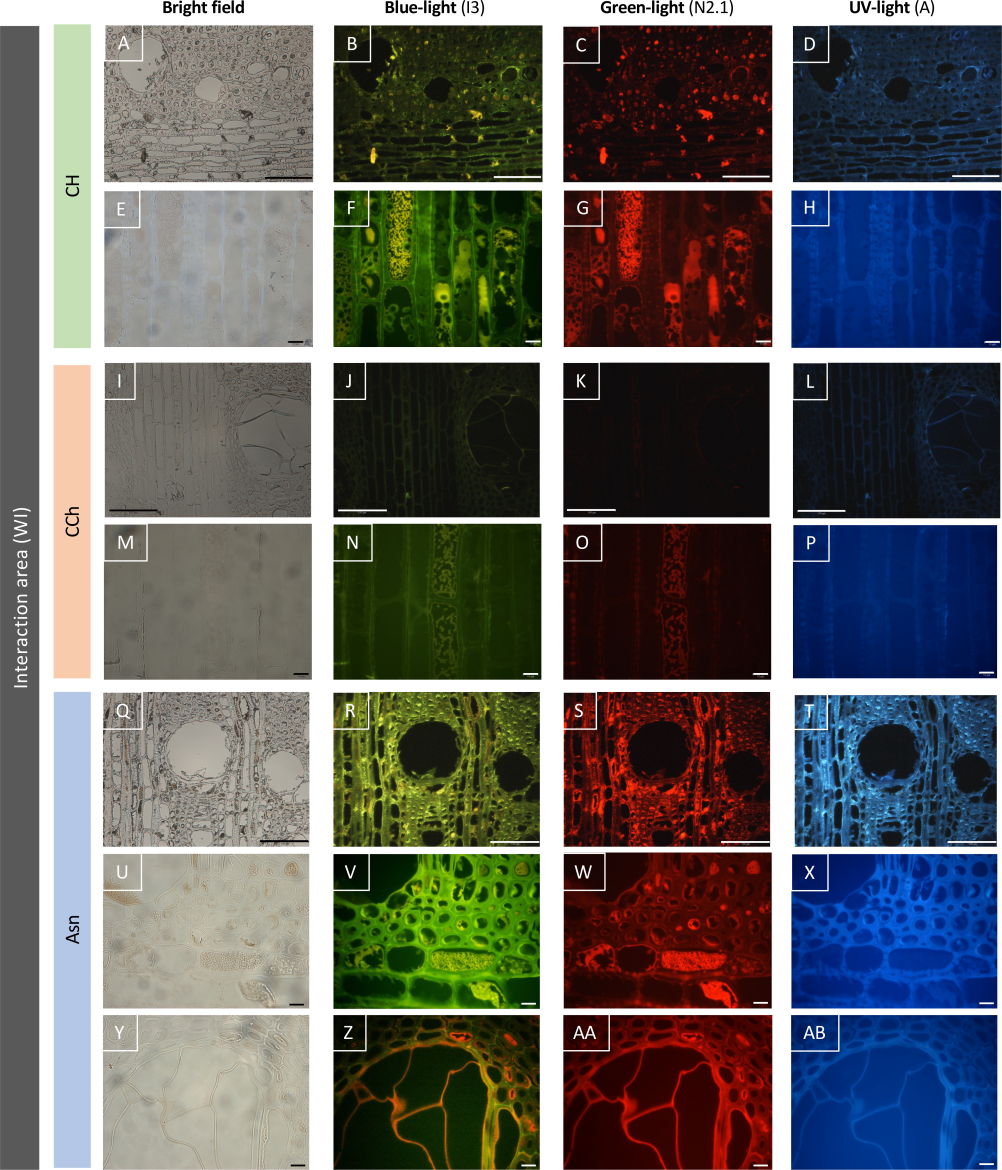
Observation of the autofluorescence of woody tissues sampled in the interaction area (WI), according to different types of excitations (blue, green, or UV light) revealed by epifluorescence microscopy. The Asn modality **(R–T, V–X, Z–AB)** reveals an intensity of autofluorescence higher than the asymptomatic control modality **(CH, B–D, F–H)**, and the wood of the CCh modality (with chronic symptoms, **J–L, N–P**) is the least autofluorescent. Bar: 100 μm **(A–D, I–L, Q–T)** or 10 μm **(E–H, M–P, U–AB)**.

In order to study more precisely the cell types affected by these defensive processes, we then observed these same types of samples after fixation and inclusion in the LRWhite resin. The results are presented in **Figures 6** and **7**. In the case of healthy woody tissues (**Figure 6**), we observed that whatever the type of modality considered (CH, CCh, and Asn), very little autofluorescence was detected. However, it appeared that in the modality where esca-diseased grapevines were treated with Asn, the signal of autofluorescence was slightly higher (**Figures 6J–L**) than what could be detected for the other two modalities (CH and CCh vines, not treated with Asn).

In the case of woody tissues collected from the interaction area (**Figure 7**), the results were clearly discriminating. Indeed, while very little autofluorescence was detected in grapevines with esca-diseased symptoms (**Figures 7J–L, N–P**), this abundance increased considerably in the two modalities for which grapevines were asymptomatic (**Figures 7B–D, F–H** for CH modality; **Figures 7R–T, V–X, Z–AB** for Asn modality). Indeed, in the asymptomatic control vines (CH modality without leaf esca symptoms and untreated with sodium arsenite), an autofluorescence was detected in the cells of ligneous rays as well as in certain ligneous fibers. This resulted in the presence of intracellular precipitates and autofluorescence under blue (**Figure 7F**) and green (**Figure 7G**) excitations, which were more or less dispersed. Cell walls appeared also autofluorescent, suggesting the impregnation of the latter by some phenolic compounds. Concerning the modality treated with sodium arsenite (Asn), a generalized parietal autofluorescence was observed in the woody tissues (**Figures 7R–T**). It was also observed regardless of the excitation of wavelength tested, suggesting that different types of phenolic compounds might be involved in this response. As in the asymptomatic control vines (CH), an intra-cellular autofluorescence was also found, in particular for the cells of ligneous rays (**Figures 7V, W**). Finally and contrary to what was observed on untreated vines with esca leaf symptoms (CCh), the wall of the tyloses also appears fluorescent (**Figures 7Z–AB**), suggesting the establishment of a defensive barrier at this level.

As it is well known that defensive phenolic molecules, such as stilbene compounds, could be autofluorescent under UV light, our cytological and metabolomic results tended to suggest that in response to sodium arsenite, certain plant defense reactions were restored in esca-diseased vines and remained effective during the vegetative year of application. However, the biosynthesis of phenolic compounds is influenced by environmental factors and rootstock variety (Chitarra et al., 2017, Costa et al., 2020). As an example, rootstock variety impacts the scion’s defensive capacity (i.e., sap phenolic levels) and its resistance against microbial pathogens (Wallis et al., 2013). Chitarra et al. (2017) reported the influence of the rootstock genotype on defense gene upregulation and stilbene accumulation in leaves of the scion. SO4, the rootstock planted in the experimental plot of our study, was among those inducing the highest stilbene inducers. In this context, it could be important to keep in mind that our results concerning Asn effects might be the result of the combination of Asn-treatment, the rootstock (SO4 in the present study) and scion (Chardonnay) varieties, and the pruning conditions (Chablis).

## Conclusion

Grapevine dieback linked to GTDs remains a sanitary and economic problem, especially since the ban on the use of sodium arsenite (Asn). In this study, we sought to better understand the impact of Asn on the physiology of vines in wood tissues at two scales: metabolomic and histological. We showed that Asn treatment carried out in March had repercussions in the wood of vines sampled until September of the same year. By comparing wood samples taken from vines symptomatic or asymptomatic of esca disease, treated or not with Asn, and in areas of healthy or interaction tissues, we revealed that Asn impacted both metabolome and structural barriers in the plant. This was all the more marked in the tissues of the interaction area. Thereby, this work highlighted for the first time that Asn enhanced the defensive pathway of the wood, especially in the interaction area where the confrontation with fungal pathogens occurred. Thus, in addition to its direct effect against certain pathogens or microbes, Asn also appeared to be able to act as a stimulator of plant secondary metabolites including defenses in the wood. The complex mode of action of Asn remains important to describe and understand with a view to developing new control techniques (phytosanitary treatments) that mimic its effects but are eco-friendly. Taking this into consideration, our recent work on the LC2017 product based on a low copper concentration with other substances showed a similar combined effect of Asn, fungistatic activity, and plant defense elicitor (Mondello et al., 2021; Mondello et al., 2022). Similarly, the combination of biocontrol agents to achieve both effects is possible (Leal et al., 2022). Nevertheless, the level of protection is lower compared to that of Asn, and the development of the best combination should be followed.

## Data availability statement

The original contributions presented in the study are included in the article/**Supplementary Material**, further inquiries can be directed to the corresponding author/s.

## Author contributions

MA, FF, PL, and ST contributed to the experimental design. PL performed the Asn treatments in the vineyard. FF, PL, LJ, ST, and JV sampled the wood of vines for metabolomic analysis. LJ and ST also sampled the wood of vines for cytological approaches. ST managed the cytological approaches and the processing of corresponding data. ST and LJ performed the macroscopic analyses in collaboration with the DImaCell Imaging Center. ST performed the scanning electron microscopic (with or without an X-ray detector) analyses in collaboration with the DImaCell Imaging Center. ST and AD performed the epifluorescence microscopic analysis. MA, CL-G, and CR-G managed the metabolomic approach and the processing of corresponding data. CL-G, CR-G, and MH performed the statistical analysis, and PS-K supervised the analysis. MA, FF, CL-G, and ST contributed to the draft of the manuscript. MA and FF managed the manuscript, and FF led the project. All authors contributed to the article and approved the submitted version.

## Supplementary material

Supplementary Figure 1 Observations of woody tissues sampled in the interaction area (WI) of Asn modality, by scanning microscopy coupled with X-ray microanalysis. By this method, we obtained simultaneously surface images (by scanning microscopy; **A, C, E, G**) and elemental composition information from a sample **(B, D, F, H)**. The elements detected in the analyzed spectra were expressed in atomic percentages **(B, D, F, H)**. This analysis revealed that no trace of Asn could be detected at this time and resolution.
