# Supplementary figures and images for: Sodium arsenite-induced changes in the wood of esca-diseased grapevine at cytological and metabolomic levels
Source: Front Plant Sci. 2023 Apr 11;14:1141700. doi: 10.3389/fpls.2023.1141700 (PMC10173745; doi:10.3389/fpls.2023.1141700)

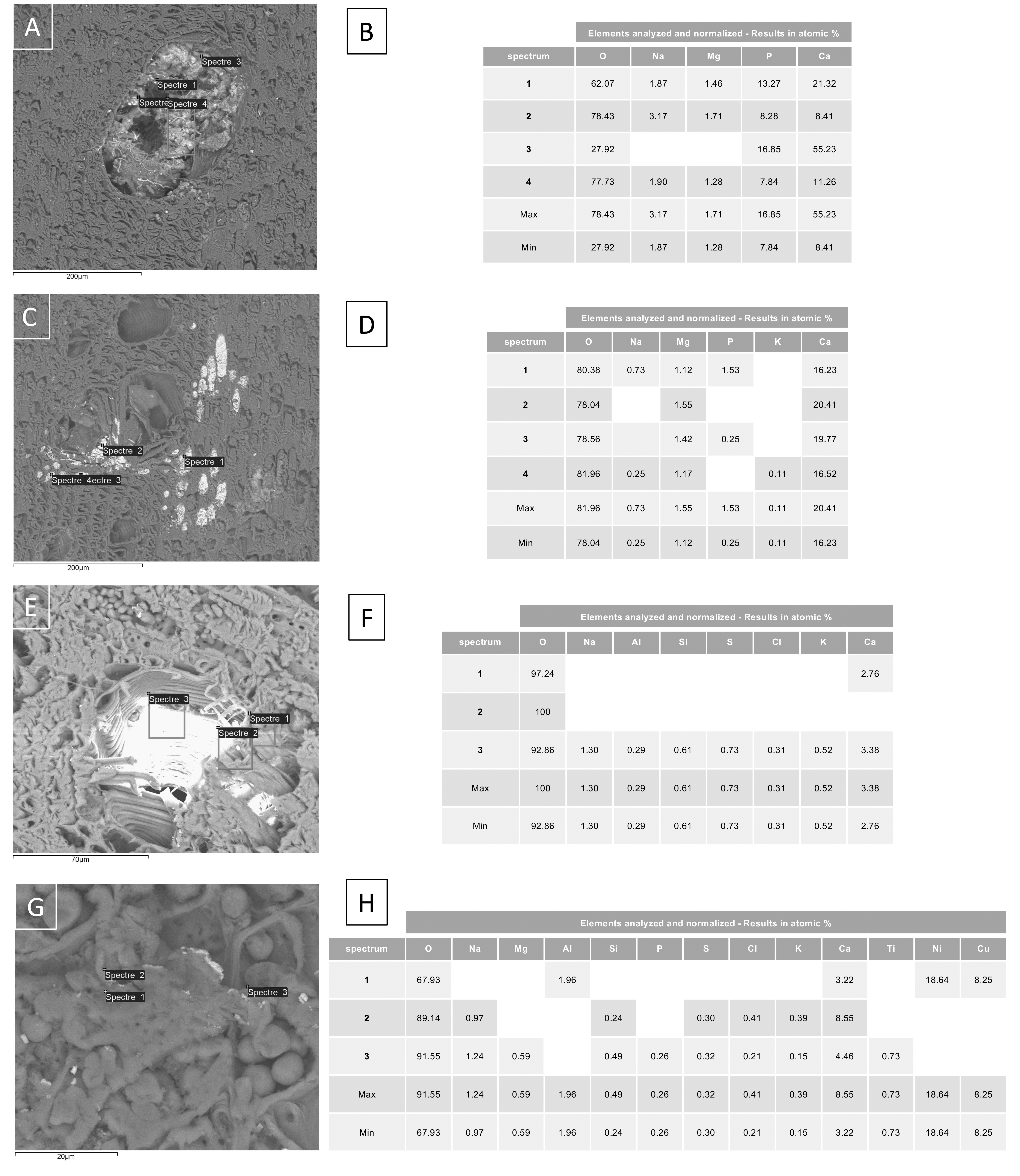

Supplement: Supplementary Figure 1 — Observations of woody tissues sampled in the interaction area (WI) of Asn modality, by scanning microscopy coupled with X-ray microanalysis. By this method, we obtained simultaneously surface images (by scanning microscopy; A, C, E, G) and elemental composition information from a sample (B, D, F, H). The elements detected in the analyzed spectra were expressed in atomic percentages (B, D, F, H). This analysis revealed that no trace of Asn could be detected at this time and resolution. [file Image_1.tif]
