# Supplementary material for: Sodium arsenite-induced changes in the wood of esca-diseased grapevine at cytological and metabolomic levels
Source: Front Plant Sci. 2023 Apr 11;14:1141700. doi: 10.3389/fpls.2023.1141700 (PMC10173745; doi:10.3389/fpls.2023.1141700)
Supplement: Supplementary file 2 [file DataSheet_1.docx]

Supplemental material. List of abbreviations used in the manuscript.

- Asn: Vines treated with sodium arsenite and without Esca foliar symptoms (Asn = Arsenite)
- CH: Vines not treated with sodium arsenite and without Esca foliar symptom (CH = Control Healthy);
- CCh: Vines not treated with sodium arsenite, expressing chronic Esca foliar symptoms (CCh = Control Chronical);
- CA: Vines not treated with sodium arsenite, expressing apoplectic foliar symptoms (CA = Control Apoplectic);
- WH: Healthy wood, without necrosis;
- WS: Streaking wood, showing very localized (punctual) brown-black necrosis points;
- WI: Wood from the interaction area between unaltered and altered wood;
- GTD: Grapevine Trunk Disease;
- cv: cultivar;
- BBCH: Biologische Bundesanstalt, Bundessortenamt und CHemische Industrie;
- FT-ICR MS: Fourier Transform - Ion Cyclotron Resonance Mass Spectrometry;
- LSSIM: Leaf Stripe Symptoms-Inducing Molecules.
