# Supplementary material for: Sodium arsenite-induced changes in the wood of esca-diseased grapevine at cytological and metabolomic levels
Source: Front Plant Sci. 2023 Apr 11;14:1141700. doi: 10.3389/fpls.2023.1141700 (PMC10173745; doi:10.3389/fpls.2023.1141700)
Supplement: Supplementary file 3 [file Table_1.docx]

| **Metabolites** | ***m/z*** | **Formula** |
| --- | --- | --- |
| *Fungal toxins* |  |  |
| OH-tyrosol | 153.05574 | C_8_H_10_O_3_ |
| Terremutin | 155.03499 | C_7_H_8_O_4_ |
| Mellein | 177.05572 | C_10_H_10_O_3_ |
| Scopoletin | 191.03500 | C_10_H_8_O_4_ |
| OH-mellein (Scytalone) | 193.05063 | C_10_H_10_O_4_ |
| 6-Methoxymellein | 207.06630 | C_11_H_12_O_4_ |
| *cis*-4-hydroxy-scytalone | 209.04554 | C_10_H_10_O_4_ |
| Tyrosol 4-sulfate | 217.01766 | C_8_H_10_O_5_S |
| Resveratrol-sulfate | 307.02819 | C_14_H_12_O_6_S |
| OH-tyrosol 1-O-glucoside | 315.10850 | C_14_H_20_O_8_ |
| Dimethylallyl-Scopoletin | 421.15040 | C_15_H_16_O_4_ |
|  |  |  |
| *Plant secondary metabolites* |  |  |
| Salicylic acid | 137.02443 | C_7_H_6_O_3_ |
| Methyl salicylate | 151.04007 | C_8_H_8_O_3_ |
| Caffeic acid | 179.03498 | C_9_H_8_O_4_ |
| Resveratrol | 227.07136 | C_14_H_12_O_3_ |
| dihydro-resveratrol | 229.08704 | C_14_H_14_O_3_ |
| Oxy-resveratrol | 243.06630 | C_14_H_12_O_4_ |
| Daidzein | 253.05068 | C_15_H_10_O_4_ |
| Linoleic acid | 279.23298 | C_18_H_32_O_2_ |
| Ellagic acid | 300.99901 | C_14_H_6_O_8_ |
| Quercetin | 301.03537 | C_15_H_10_O_7_ |
| Caftaric acid | 311.04081 | C_13_H_12_O_9_ |
| Galloyl-glucose | 331.06701 | C_13_H_16_O_10_ |
| Piceid | 389.12415 | C_20_H_22_O_8_ |
| Resveratrol-O-glucuronide | 403.10345 | C_20_H_20_O_9_ |
| Methyl-resveratrol-glucoside | 403.13983 | C_21_H_24_O_8_ |
| Astringin | 405.11909 | C_20_H_22_O_9_ |
| epsilon-viniferin | 453.13440 | C_28_H_22_O_6_ |
| Resveratrol-glucoside-sulfate | 469.08097 | C_20_H_22_O_11_S |
| Quercetin-O-glucuronide | 477.06748 | C_21_H_18_O_13_ |
| Resveratrol-galloylglucoside | 577.11177 | C_27_H_26_O_12_ |
| delta-viniferin-glucoside | 615.18723 | C_34_H_32_O_11_ |
| epsilon-viniferin-diglucoside | 777.23995 | C_40_H_42_O_16_ |

**Supplementary Table S1**. List of targeted fungal toxins and plant secondary metabolites with corresponding *m/z* and raw formula.
