# Supplementary material for: Sodium arsenite-induced changes in the wood of esca-diseased grapevine at cytological and metabolomic levels
Source: Front Plant Sci. 2023 Apr 11;14:1141700. doi: 10.3389/fpls.2023.1141700 (PMC10173745; doi:10.3389/fpls.2023.1141700)
Supplement: Supplementary file 4 [file Table_2.docx]

**Supplementary Table S2**. Count of significant *m/z* and metabolites during the analysis process in wood interaction area. Analysis process started from initial significant *m/z* lists (*P* < 0.05) able to distinct between two samples groups (Perseus software). These lists generated raw formulas assigned by NetCalc software. Then formulas were applied to van Krevelen parameters to determine C/H/N/O/N/S atomic proportion validations and chemical family classification. In parallel, raw formulas were submitted to MassTrix queries for compounds identification and resulting annotations led to manual revisions and curation of formula redundancies. Lists were compared to indicate the number and proportions of common compounds.

|  | WI-Asn *versus* WI-CH | WI-Asn *versus* WI-CCh | Common  (% identical) |
| --- | --- | --- | --- |
| Significant *m/z* (T-Test) | 1132 | 1162 | 1121 (98%) |
| Raw formulas | 1071 | 1097 | 1061 (98%) |
| van Krevelen formulas (C/H/N/O/P) | 1057 | 1087 | 1051 (98%) |
| Non-redundant formulas | 1037 | 1067 | 999 (95%) |
| Annotated compounds | 546 | 557 | 424 (75%) |
