# Supplementary material for: Sodium arsenite-induced changes in the wood of esca-diseased grapevine at cytological and metabolomic levels
Source: Front Plant Sci. 2023 Apr 11;14:1141700. doi: 10.3389/fpls.2023.1141700 (PMC10173745; doi:10.3389/fpls.2023.1141700)
Supplement: Supplementary file 5 [file Table_3.docx]

**Supplementary Table S3**: Lists of the TOP20 most regulated compounds in wood interaction area (WI) after sodium arsenite treatment (Asn). Compounds were sorted according to their abundance differences calculated *versus* Asn-treated grapevines (indicated in squared columns). Negative values mean less abundant in Asn-samples and positive ones mean accumulation in Asn-WI samples compared to Healthy (CH, **Table A**) or Chronic diseased (CCH, **Table B**)-WI samples. Only compounds identified by KEGG are listed. Identification number (ID), *m/z* and error (in ppm), raw formula, and functional category (Cat.: phytochemicals: Ph; lipid-like: Lp; carbohydrates: Cb, amino sugar-like: AmS and unmatched: NM) are indicated and also when compounds were common in the two comparisons Asn *vs* CH and Asn *vs* CCH.

**A)** TOP20 list of differentially accumulated compounds between WI-Asn and WI-CH samples.

| **ID** | ***m/z*** | **KEGG mass** | **error ppm** | **common or specific** | **WI-Asn *vs***  **WI-CH** | **Cat.** | **KEGG formula** | **KEGG_name** |
| --- | --- | --- | --- | --- | --- | --- | --- | --- |
| **23079** | 443.09848 | 444.105650 | -0.240353 | CH | -1.29 | Ph | C_22_H_20_O_10_ | Granaticin |
| **15851** | 307.059014 | 308.066107 | -0.598257 | CCh CH | -1.13 | Lp | C_12_H_16_O_7_ | Arbutin; α-Arbutin |
| **13316** | 273.038274 | 274.045372 | -0.655220 | CCh CH | -1.06 | Cb | C_8_H_14_O_8_ | 3-Deoxy-D-manno-octulosonate |
| **13692** | 279.042940 | 280.050062 | -0.554754 | CCh CH | -0.52 | Ph | C_14_H_12_O_4_ | Piceatannol; Wyerone acid; Eriobofuran; Fulvoplumierin; Oxyresveratrol; Methylstyrylpyron; 2,2'-Dihydroxy-4-methoxybenzophenone; Suberenone; (2E,11Z)-Wyerone acid; Graveolone; 9,10-Dihydro-2,3,5,7-Phenanthrenetetrol; (R)-Apiumetin |
|  |  |  |  |  |  |  |  |  |
| **17992** | 333.191866 | 334.199155 | 0.037216 | CCh CH | +2.51 | Lp | C_16_H_30_O_7_ | 2,6-Dimethyl-7-octene-1,6-diol 8-O-glucoside; *trans*-p-Menthane-7,8-diol 7-glucoside; *trans*-p-Menthane-7,8-diol 8-glucoside; 3,7-Dimethyl-5-octene-1,7-diol 1-glucoside; 3-O-α-L-rhamnopyranosyl-3-hydroxydecanoic acid |
| **21658** | 403.124547 | 404.131865 | 0.101457 | CCh CH | +2.46 | NM | C_17_H_24_O_11_ | Gardenoside; Scandoside methyl ester; Oleoside 11-methyl ester |
| **18656** | 341.133639 | 342.140884 | -0.093512 | CCh CH | +2.40 | NM | C_13_H_24_O_10_ | O^18^ isotope of Methyl-2-α-L-fucopyranosyl-β-D-galactoside |
| **25843** | 536.112720 | 537.119937 | -0.111917 | CCh CH | +2.34 | Ph | C_24_H_24_O_14_ | C^13^ isotope of Laricitrin 3-(6''-acetylglucoside) |
| **20486** | 375.129670 | 376.136950 | 0.007464 | CCh CH | +2.33 | NM | C_16_H_24_O_10_ | Loganate; 7-Epiloganic acid; (1RS,2RS)-Guaiacylglycerol 1-glucoside; (1x,2x)-Guaiacylglycerol 2-glucoside; (1x,2x)-Guaiacylglycerol 3-glucoside |
| **17257** | 325.114028 | 326.121300 | -0.013841 | CCh CH | +2.31 | Cb | C_12_H_22_O_10_ | 2-O-α-L-Rhamnopyranosyl-D-glucopyranose; Robinobiose; Rutinose; Scillabiose; β-D-Galactosyl-(1->4)-L-rhamnose; 2-O-α-L-Fucopyranosyl-galactose; 3-O-α-L-Fucopyranosyl-D-glucose; Neohesperidose; 6-O-β-D-Fructofuranosyl-2-deoxy-D-glucose |
| **19457** | 353.145301 | 354.152600 | 0.062864 | CCh CH | +2.29 | Cb | C_14_H_26_O_10_ | 2-((6-O-(β-D-apiofuranosyl) β -D-glucopyranosyl)oxy)propane; Isopropyl apiosylglucoside |
| **10517** | 219.08742 | 220.094690 | -0.029669 | CCh CH | +2.29 | Pp | C_8_H_14_N_2_O_5_ | O^18^ isotope of γ-L-Glutamyl-D-alanine |
| **19116** | 347.113900 | 348.120905 | -0.784469 | CCh CH | +2.28 | Ph | C_18_H_20_O_7_ | Machaerol C; 6,8,2'-Trihydroxy-7,3',4'-trimethoxyisoflavan |
| **11762** | 245.139448 | 246.146725 | 0.000816 | CCh CH | +2.28 | Lp | C_12_H_22_O_5_ | 3-Hydroxydodecanedioic acid; Dibutyl malate |
| **23230** | 447.129633 | 448.136950 | 0.089907 | CCh CH | +2.27 | Ph | C_22_H_24_O_10_ | Sakuranin; Isosakuranin; 7-Hydroxy-8-O-methylaloin B; Puddumin A; Chalconosakuranetin; Aromadendrin 4'-methyl ether 7-rhamnoside; Piperenol C; 5-Hydroxy-3,6,7,8,3',4',5'-heptamethoxyflavone; 3'-Hydroxy-3,5,6,7,8,4',5'-heptamethoxyflavone; Agehoustin C; Haplanthin; Isosakuranin; Puddumin B; Isosakuranetin 5-O-glucoside; Alhagitin; Dihydrowogonin 7-O-glucoside; 6,7-Dihydroxy-5-methoxyflavanone 7-O-glucoside; Androechin; 4,2',4'-Trihydroxy-6'-methoxychalcone 4-glucoside; Helichrysin; Homobutein 4-glucoside; Homobutein 4'-O-glucoside; Poriolin; Hesperetin 7-O-rhamnoside |
| **10694** | 224.10089 | 225.108187 | 0.091477 | CCh CH | +2.27 | Lp | C_12_H_16_O_4_ | C^13^ isotope of Aspidinol |
| **27252** | 623.255657 | 624.262940 | 0.009627 | CCh CH | +2.27 | Lp | C_27_H_44_O_16_ | Kanokoside D |
| **24007** | 469.114092 | 470.121300 | -0.147725 | CCh CH | +2.27 | Ph | C_24_H_22_O_10_ | Pongamoside B; Pongamoside C |
| **26154** | 553.192674 | 554.199945 | -0.011388 | CCh CH | +2.27 | Ph | C_26_H_34_O_13_ | Osthenol-7-O-β-D-gentiobioside; Marmesin rutinoside |
| **19661** | 357.098051 | 358.105255 | -0.204986 | CCh CH | +2.27 | Ph | C_19_H_18_O_7_ | Gardenin B; 5-Hydroxy-2-(4-methoxyphenyl)-6,7,8-trimethoxy-4H-1-benzopyran-4-one; 5-Hydroxy-4',6,7,8-tetramethoxyflavone; Demethyltangeretin |
| **15731** | 305.073232 | 306.080316 | -0.632963 | CCh CH | +2.27 | NM | C_12_H_18_N_2_O_3_S | Tolbutamide |
| **20940** | 385.165631 | 386.172940 | 0.084120 | CCh CH | +2.27 | Ph | C_22_H_26_O_6_ | Burseran; (+)-Eudesmin; Porson; Isogingerenone B; Gingerenone B; 3,5-Di-O-methyl-8-prenylafzelechin-4β-ol; 4,5-Di-O-methyl-8-prenylafzelechin-4β-ol |
| **15882** | 307.191491 | 308.198760 | -0.027019 | CCh CH | +2.27 | Lp | C_18_H_28_O_4_ | 5-O-Methylembelin |
| **24011** | 469.123764 | 470.131032 | -0.020037 | CCh CH | +2.27 | Ph | C_21_H_24_O_12_ | O^18^ isotope of Catechin-4-ol 3-O-β-D-galactopyranoside |

**B)** TOP20 list of differentially accumulated compounds between WI-Asn and WI-CCh samples.

| **ID** | ***m/z*** | **KEGG mass** | **error ppm** | **common or specific** | **WI-Asn - WI-CCh** | **Cat.** | **KEGG formula** | **KEGG_name** |
| --- | --- | --- | --- | --- | --- | --- | --- | --- |
| **13316** | 273.038274 | 274.045372 | -0.655220 | CCh CH | -2.55 | Cb | C_8_H_14_O_8_ | 3-Deoxy-D-manno-octulosonate |
| **10112** | 208.985855 | 209.992942 | -0.910110 | CCh | -2.41 | Ph | C_6_H_6_O_6_ | *cis*-Aconitate; *trans*-Aconitate; Dehydroascorbate; Dehydroascorbic acid |
| **19626** | 356.113940 | 357.121239 | 0.061778 | CCh | -2.36 | Ph | C_19_H_19_NO_6_ | Gravacridonetriol |
| **15851** | 307.059014 | 308.066107 | -0.598257 | CCh CH | -2.35 | Lp | C_12_H_16_O_7_ | Arbutin; α-Arbutin |
| **8650** | 128.035324 | 129.042594 | -0.053891 | CCh | -2.23 | Pp | C_5_H_7_NO_3_ | 4-Oxoproline; 4-Oxo-L-proline |
| **18251** | 338.088133 | 339.095419 | 0.026029 | CCh | -1.93 | Ph | C_15_H_17_NO_8_ | 6-Hydroxy-5-methoxyindole glucuronide; 5-Hydroxy-6-methoxyindole glucuronide |
| **24067** | 470.151528 | 471.158809 | 0.008083 | CCh | -1.91 | AmS | C_17_H_29_NO_14_ | α-N-Acetylneuraminyl-2,3-β-D-galactosyl |
| **15973** | 309.102109 | 310.109493 | 0.347134 | CCh | -1.71 | Ph | C_19_H_16_O_4_ | O^18^ isotope of Warfarin |
| **8662** | 129.019343 | 130.026610 | -0.075958 | CCh | -1.70 | Ph | C_5_H_6_O_4_ | 2,5-Dioxopentanoate; Itaconate; Mesaconate; Acetylpyruvate; (E)-Glutaconate; 2-Methylmaleate; 4,5-Dioxopentanoate; Methyl hydrogen fumarate; 2-Hydroxyglutaric acid lactone |
| **22805** | 437.122077 | 438.129102 | -0.575583 | CCh | -1.63 | Cb | C_18_H_26_O_10_ | benzyl alcohol 6-O-β-D-xylopyranosyl-β-D-glucopyranoside; benzyl 6-O-β-D-apiofuranosyl-β-D-glucopyranoside; Benzyl β-primeveroside; Benzyl O-[arabinofuranosyl-(1->6)-glucoside] |
| **25847** | 536.177396 | 537.184629 | -0.081130 | CCh | -1.56 | Ph | C_25_H_31_NO_12_ | 4-Demethylsimmondsin 2'-(E)-ferulate; 3-Demethylsimmondsin 2'-(Z)-ferulate |
| **16908** | 320.062288 | 321.069599 | 0.104980 | CCh | -1.55 | NM | C_11_H_15_NO_10_ | β-Citryl-L-glutamic acid |
| **9551** | 188.092832 | 189.100109 | -0.001063 | CCh | -1.48 | Lp | C_8_H_15_NO_4_ | Castanospermine; (2S)-2-{[1-(R)-Carboxyethyl]amino}pentanoate; Alexine; Australine;  2-(Butylamido)-4-hydroxybutanoic acid; (2S)-2-{[1-(R)-carboxyethyl]amino}pentanoate;  N-Methylcalystegine B2 |
| **21423** | 395.111391 | 396.118537 | -0.330793 | CCh | -1.45 | NM | C_16_H_24_O_10_ | 7-Deoxyloganate; 8-Epideoxyloganic acid; 1-(3,4-Dimethoxyphenyl)-1,2-ethanediol 1-O β-D-glucoside; 1-(3,4-Dimethoxyphenyl)-1,2-ethanediol 2-O-β-D-glucoside; Deoxyloganic acid; 2-(4-Hydroxy-3,5-dimethoxyphenyl)ethanol 4'-glucoside; 2'-Methoxy-3-(2,4-dihydroxyphenyl)-1,2-propanediol 4'-glucoside |
| **13692** | 279.042940 | 280.050062 | -0.554754 | CCh CH | -1.32 | Ph | C_14_H_12_O_4_ | Piceatannol; Wyerone acid; Eriobofuran; Fulvoplumierin; Oxyresveratrol; Methylstyrylpyron; 2,2'-Dihydroxy-4-methoxybenzophenone; Suberenone; (2E,11Z)-Wyerone acid; Graveolone; 9,10-Dihydro-2,3,5,7-Phenanthrenetetrol; (R)-Apiumetin |
| **28110** | 701.112491 | 702.119722 | -0.065182 | CCh | -1.27 | Ph | C_29_H_30_O_18_ | (3''-Apiosyl-6''-malonyl)astragalin |
| **15913** | 308.098671 | 309.105984 | 0.115547 | CCh | -1.12 | AmS | C_11_H_19_NO_9_ | N-Acetylneuraminate; O-Acetylneuraminic acid; N-Acetyl-α-neuraminate; N-Acetyl-β-neuraminate; N-Acetylneuraminic acid; N-Acetyl-α-neuraminic acid |
| **21573** | 401.075733 | 402.083201 | 0.476219 | CCh CH | -0.47 | Ph | C_21_H_22_O_3_ | Isoderricin A; Derricin |
|  |  |  |  |  |  |  |  |  |
| **16622** | 315.181308 | 316.188590 | 0.015864 | CCh CH | +2.76 | Lp | C_16_H_28_O_6_ | Neryl glucoside; (S)-α-Terpinyl glucoside; D-Linalool 3-glucoside; 5(6)-Butyl-1,4-dioxan-2-one; Perilloside C; Menthol-glucoronide |
| **20954** | 385.332328 | 386.339610 | 0.013495 | CCh CH | +2.64 | Lp | C_23_H_46_O_4_ | DG(18:0e/2:0/0:0); MG(0:0/20:0/0:0); MG(20:0/0:0/0:0); MG(20:0/0:0/0:0)[rac] |
| **22028** | 413.363587 | 414.370910 | 0.110314 | CCh CH | +2.58 | Lp | C_25_H_50_O_4_ | MG(0:0/22:0/0:0); MG(22:0/0:0/0:0); Glyceryl behenate |
| **20362** | 371.243866 | 372.251190 | 0.125793 | CCh CH | +2.57 | Lp | C_20_H_36_O_6_ | 8,8a-Deoxyoleandolide; 19R-hydroxy-PGF1α; 13,14-dihydro-19R-hydroxyPGE1; TXB1; 8,8a-Deoxyoleandolide |
| **17724** | 330.069956 | 331.077282 | 0.147847 | CCh CH | +2.57 | Ph | C_17_H_14_O_7_ | C^13^ isotope of 3',4',5-Trihydroxy-3,7-dimethoxyflavone; 3,7-Di-O-methylquercetin |
| **20364** | 371.316637 | 372.323960 | 0.123345 | CCh CH | +2.54 | Lp | C_22_H_44_O_4_ | 13,14-dihydroxy-docosanoic acid |
| **9792** | 197.118317 | 198.125595 | 0.004566 | CCh CH | +2.54 | NM | C_22_H_36_O_6_ | O^18^ isotope of Fasoracetam; NS 105 |
| **20864** | 383.207510 | 384.214805 | 0.046972 | CCh CH | +2.53 | Lp | C_20_H_32_O_7_ | 20-Trihydroxy-leukotriene-B4; Cinnzeylanol |
| **17329** | 325.201924 | 326.209325 | 0.380994 | CCh CH | +2.52 | Lp | C_18_H_30_O_5_ | 2,3-Dinor-8-iso prostaglandin F2α; 2,3-Dinor-8-iso PGF2α |
| **19577** | 355.118671 | 356.125990 | 0.117707 | CCh CH | +2.51 | Ph | C_20_H_20_O_6_ | Kievitone; Plaunol B; Cubebin; Coniferyl ferulate; Leachianone G; (+)-piperitol; norsolorinate anthrone; Xanthoxylol; Cubebin; Licoagrodione; Licofuranone; 5-Deoxykievitol; Kinobeon A; 1,8-Dihydroxy-3,5-dimethoxy-2-prenylxanthone; Tomentosanol D; Sigmoidin B; Flowerone; 2'-Prenyleriodictyol; 8-Prenyleriodictyol; 6-Prenyleriodictyol; Leachianone G; Diphysolone; Kalmiatin; Ramosismin; Monotesone A; Laurifolin (flavonoid); Kenusanone J; Phellodensin D; 2',4'-Dihydroxy-2''-(1-hydroxy-1-methylethyl)dihydrofuro[2,3-h]flavanone; Brosimacutin F; Brosimacutin G; (2S)-5,7,3',4'-Tetrahydroxy-6-(1,1-dimethylallyl)flavanone |
| **24014** | 469.129337 | 470.136555 | -0.126618 | CCh CH | +2.46 | Ph | C_28_H_22_O_7_ | Didymocalyxin B |
| **14911** | 295.191478 | 296.198760 | 0.016938 | CCh CH | +2.43 | Lp | C_17_H_28_O_4_ | 10-Deoxymethynolide |
| **26579** | 577.447288 | 578.454640 | 0.130748 | CCh CH | +2.41 | Lp | C_35_H_62_O_6_ | Squamocin K; Asimilobin; Rollidecin C; Isomurisolenin; Corossolone; Stigmastanyl glucoside |
| **24698** | 487.139874 | 488.147120 | -0.063021 | CCh CH | +2.40 | Ph | C_28_H_24_O_8_ | Bisosthenon B; Khelmarin D |
| **23377** | 451.139812 | 452.147120 | 0.068715 | CCh CH | +2.39 | Ph | C_25_H_24_O_8_ | Urdamycinone B; Landomycin H; Epimedokoreanin A; (9R,10S)-rel-(-)-9,10-bis(Acetyloxy)-9,10-dihydro-5-methoxy-8,8-dimethyl-2-phenyl-4H,8H-benzo[1,2-b:3,4-b']dipyran-4-one |
| **26784** | 591.171858 | 592.179210 | 0.126697 | CCh CH | +2.37 | Ph | C_28_H_32_O_14_ | 1,2-Bis-O-sinapoyl-β-D-glucoside; Isoswertisin 2''-rhamnoside; 7-O-methylvitexin 2''-O-β-L-rhamnoside; ax-4''-Hydroxy-3-'-methoxymaysin; Liquiritigenin 4'-[3-acetylapiosyl-(1->2)-glucoside]; Isomargaritene; Isowertin 2''-rhamnoside; Margaritene; Kaempferide 3,7-dirhamnoside; Flavocummelin; Fagovatin; Isoswertisin 4'-O-rhamnoside; Isocytisoside 3''-O-α-L-rhamnopyranoside; Isomargaritene; Swertisin 2''-O-rhamnoside; Cytisoside 3''-O-β-D-rhamnopyranoside; Margaritene; Isoswertisin 2''-O-rhamnoside; Torosaflavone B 3'-O-β-D-glucopyranoside; Cannabin; Retusin 7-O-neohesperidoside; Baicalein 6-methyl ether 7-glucosyl-(1->3)-rhamnoside; Biochanin A 7-O-rutinoside; Acacetin 7-rutinoside; Acacetin 7-neohesperidoside; Liquiritigenin 7-(3-acetylapiofuranoside)-4'-glucoside; Liquiritigenin 4'-[3-acetylapiosyl-(1->2)-glucoside]; 5,4'-Dimethoxyflavone 7-xylosyl-(1->6)-glucoside; Isomollupentin 7,4'-dimethyl ether 2''-O-glucoside; Formononetin 7-O-laminaribioside; 2''-O-α-L-Rhamnosyl-6-C-fucosyl-3'-methoxyluteoiin |
| **20476** | 375.087451 | 376.094690 | -0.101310 | CCh CH | +2.35 | Ph | C_22_H_16_O_6_ | Resistomycin; Naphthoherniarin; Dehydromillettone |
| **10214** | 211.133977 | 212.141245 | -0.042627 | CCh CH | +2.34 | Lp | C_12_H_20_O_3_ | Cucurbic acid |
| **25843** | 536.112720 | 537.119937 | -0.111917 | CCh CH | +2.34 | Ph | C_24_H_24_O_14_ | C^13^ isotope of Laricitrin 3-(6''-acetylglucoside) |
| **24106** | 471.348040 | 472.355260 | -0.121566 | CCh CH | +2.34 | Lp | C_30_H_48_O_4_ | Gratiogenin; Echinocystic acid; Alphitolic acid; Crataegolic acid; Siaresinol; Sumaresinol; Alisol B; 2-hydroxyoleanolate; hederagenin; Lucidumol A; Queretaroic acid; Priverogenin A; (3β,23xi)-3,23-Dihydroxycycloart-24-en-26-oic acid; Pomolic acid; 20β-Hydroxyursolic acid; Azukisapogenol; δ-Maslinic acid; Sebiferenic acid; Momordicin I; Ganoderic acid U; Ganodermanontriol; Epoxyganoderiol A; (3b,22S,24E)-3,22-Dihydroxycycloart-24-en-26-oic acid; 27-Hydroxyisomangiferolic acid; Albigenic acid; Rubitic acid; Ganoderiol E; 3α-Corosolic acid; spinosic acid A; 16-Oxolycoclavanol; 26,27-diethyl-1α,25-dihydroxy-20,21-didehydro-23-oxavitamin D3 / 26,27-diethyl-1α,25-dihydroxy-20,21-didehydro-23-oxacholecalciferol |
